# Identification of QTLs involved in destemming and fruit quality for mechanical harvesting of New Mexico pod–type green chile

**DOI:** 10.3389/fpls.2024.1357986

**Published:** 2024-07-01

**Authors:** Franchesca Ortega, Theresa Hill, Allen Van Deynze, Armando Garcia-Llanos, Stephanie Walker

**Affiliations:** ^1^ Department of Extension Plant Sciences, New Mexico State University, Las Cruces, NM, United States; ^2^ Seed Biotechnology Center, University of California, Davis, Davis, CA, United States

**Keywords:** *Capsicum annuum*, pepper, Chile pepper, destemming, fruit size, mechanical harvest

## Abstract

**Introduction:**

Domestic production of pepper (Capsicum spp.) is shrinking while demand within the US is growing. Lack of availability and cost of labor often present an obstacle for domestic producers both practically and economically. As a result, switching to harvesting peppers mechanically is anticipated as a key strategy to help domestic producers compete in the international market. Mechanical harvest efficiency can be improved through breeding. One important trait that mechanical harvest compatible material should have is an easy destemming trait: low force separation of the pedicel and calyx from the fruit.

**Methods:**

To detect the genetic sources underlying a novel easy destemming trait for the purpose of future breeding efforts in New Mexico pod-type green chile, we performed QTL analysis on three F2:F3 populations, coming from three New Mexico pod-type varieties: ‘NuMex Odyssey,’ ‘NuMex Iliad,’ and ‘NuMex Joe E. Parker,’ each crossed with a parent with an easy destemming trait: MUC14. Genotyping was done through genotyping by sequencing (GBS) and phenotyping was done for destemming and fruit trait measurements. Correlations between measurements were found through the R package hmisc and QTL analysis was done through R/qtl.

**Results:**

A strong relationship was seen between destemming and aspects of fruit morphology, particularly, destemming force and fruit width (Pearson’s correlation coefficient r=0.75). Major QTLs for destemming and fruit size were discovered. Of these, the largest destemming force QTLs for all populations (PVE=34.5-69.9%) were on chromosome 10, and in two populations QTLs for destemming force were found on chromosome 3 (Percent Variance Explained (PVE)=10.7-18.8%). Fruit size-related QTLs in all populations colocalized in these same areas on chromosomes 3 and 10.

**Discussion:**

This suggests that fruit shape may be genetically linked to destemming, and breeders interested in selecting for easy destemming pepper will also have to pay attention to fruit size and shape.

## 1 Introduction

In New Mexico, chile pepper (*Capsicum* spp.) is an economically and culturally important crop. The state is a major hot pepper producer, accounting for 82.8%, 80.2%, and 83.7% of the total US chile pepper harvest reported for 2020, 2021, and 2022, respectively (USDA and NASS, 2023). It is also the origin of a type of pepper that bears the state’s name: New Mexico pod–type pepper (this pod shape is sometimes also called Anaheim pepper). Long, smooth, and mild to moderately hot, New Mexico pod–type peppers were first developed and released by the horticulturalist Fabian Garcia at New Mexico State University in the early 20th century (Coon et al., 2008). Today, New Mexico pod–type peppers are an integral part of “Mexican” cuisine in the American southwest, with many varieties (New Mexico 6-4, NuMex Big Jim, and NuMex Joe E. Parker) being commonly grown both within New Mexico and in nearby states (Bosland and Walker, 2014).

Both red (ripe) and green (un-ripe) chile are grown and consumed, but, whereas the red chile crop is mostly mechanically harvested, the green chile crop is still entirely hand-harvested: a task that presents challenges for producers within the US (Wall et al., 2003; Walker and Funk, 2014). An aging workforce, changing immigration laws, and labor reforms affect labor availability, making it uncertain that there will be a workforce available at harvest time. In addition, it is estimated that manual harvesting is responsible for up to 50% of the cost of domestic chile production (Hawkes et al., 2008). Because of this, US pepper production is not increasing alongside demand. Instead, the total acreage of chile pepper grown within the US is shrinking, whereas imports from countries with cheaper and more readily available labor are increasing (Gandonou and Waliczek, 2013; Lillywhite and Tso, 2021). As a result, the implementation of mechanical harvesting for green chile is anticipated as a key strategy to help domestic pepper producers compete in an international market.

Field trials indicate that successful mechanical harvesting in green chile requires a systems approach because the choice of harvester, field management techniques, and pepper varieties impact the efficacy of mechanical harvesting (Funk et al., 2011). For the delicate green chile crop, which deteriorates quickly if broken, the harvester chosen should be relatively gentle. Currently, the most promising harvester in this respect is an inclined double helix machine produced by Yung-Etgar (Bet-Lehem-Haglilit, Israel) (Funk and Walker, 2010). The height and growth habit of plants also affect mechanical harvesting, so field management decisions about plant spacing and transplanting vs. direct seeding that impact plant structure are relevant to the system as well (Havlik, 2021). Finally, access to plant material that is bred to have traits suitable for mechanical harvesting is critical.

There are certain plant and fruit traits that are important for mechanical harvest pepper varieties. To be compatible with the double helix harvester, plants should have upright architecture with a tall, single major stem, lack of low-lying lateral branches (sometimes called basal branches), and larger basal stem diameters (Joukhadar et al., 2018). Fruit traits like uniform ripening, thicker fruit walls (pericarp thickness), and easy to remove pedicels (easy destemming) would also be conducive to an ideal result: mechanically harvested, uniformly mature, stemless, and intact green chile.

Efforts to develop mechanical harvest–amenable plant material are at the early stages. Recently, a New Mexico pod–type variety called ‘NuMex Odyssey’ that was bred to have a main single stem and increased height to fruit set, ideal plant architecture traits for green chile mechanical harvest, was released (Walker et al., 2021). This is currently the only commercially available green chile New Mexico pod–type variety that has been developed with mechanical harvest in mind. With regard to the genetics of stem removal, a quantitative trait locus (QTL) study was recently conducted for two populations derived from a semi-domesticated pepper with a novel easy destemming trait called UCD-14, revealing several QTLs with a large effect (Hill et al., 2023). Studies such as this can be a step toward breeding through marker-assisted selection, an expedited breeding method in which individuals of a population are screened and selected on the basis of the presence of genes that confer traits of interest.

Considering this, to aid in the development of New Mexico pod–type green chile varieties that are amenable to mechanical harvest, we conducted a QTL study for three populations that came from crosses between a New Mexico pod–type variety and a semi-domesticated landrace with a novel easy destemming trait.

## 2 Materials and methods

### 2.1 Population descriptions

Three intercross populations were created, each from two parent reciprocal crosses where one parent was a green chile New Mexico pod–type variety, and the other parent was a variety with an easy destemming trait (MUC14). The three New Mexico pod–type parents were ‘NuMex Iliad,’ ‘NuMex Odyssey,’ and ‘NuMex Joe E. Parker.’ Of these, two were breeding lines developed at New Mexico State University, ‘NuMex Iliad’ and ‘NuMex Odyssey,’ to be compatible with mechanical harvesting in terms of their plant architecture. The other, ‘NuMex Joe E. Parker,’ was a line that had very few destemmed fruits harvested in mechanical harvest trials. The parent crossed with each New Mexico pod–type is MUC14. This is a line derived from a cross between a semi-domesticated Mexican landrace with small fruit and a novel destemming trait (UCD-14) and Maor that was selected for low destemming force and performance in mechanical harvest. For all three populations, the F_1_ and F_2_ generations were grown in a greenhouse at Fabian Garcia Science Center, Las Cruces, NM. The F_3_ generation was grown at the NMSU Agricultural Science Center in Los Lunas, NM. The F_3_ families were direct-seeded in five-foot plots, with two replicates per family. We aimed to collect 40 mature green fruits per plot, and we collected an average of 38 fruits from six plants per plot. The field was planted in April and harvested in September 2021.

### 2.2 Phenotyping

Five measurements were taken from each fruit collected: fruit width, fruit length, pericarp thickness, destemming score, and destemming force. The height was measured at the tallest length of the fruit, and the width was measured across the fruit body below the shoulder. Fruits were cut at the width measurement, and the pericarp thickness was measured with calipers. A representation of overall fruit size, fruit size index, was recorded as fruit width times fruit length. To measure the destemming force, a Tohnichi torque gauge (Tohnichi America Corporation, Buffalo Grove, IL, United States) with a custom metal fork attachment that enables the removal of a pedicel with a twisting motion, meant to resemble the motion of the harvester, was used (Hill et al., 2023). At the same time, a subjective rating of destemming efficiency, called “destemming score,” was taken, where the measurer rated the quality of the fruit after destemming. The scale was from 1 to 5, where 1 meant a perfectly destemmed fruit, 2 to 4 meant the stem was removed, but with increasing damage to the fruit, and 5 meant there was a failure to destem. For each measurement, an average was taken for all the fruit measurements per plot, and the average of the two replicate plots was used as the final phenotype representing the F_2_ parent.

### 2.3 DNA extraction

Young leaf tissue was taken from the parents (‘NuMex Joe E. Parker,’ ‘NuMex Odyssey,’ ‘NuMex Iliad,’ and MUC14) and F_2_ individuals from each population and collected into 2-mL screw cap tubes with 2.5-mm Zirconia/Silica beads and stored at −80°C until extraction. Before homogenization with a Precellys 24 Homogenizer (Bertin instruments Paris, France), buffer AP1 (QIAGEN, Germany) was added to the tubes. The QIAGEN DNeasy Plant Mini Kit was used to extract DNA, and the concentration and absorbance ratios were checked with a Nanodrop 2000 Spectrophotometer (Thermo Fisher Scientific, Waltham, MA US). RNA contamination was checked using gel electrophoresis.

### 2.4 Genotyping by sequencing

Libraries were constructed using the protocol described by Elshire et al. (2011), with some modifications. Instead of using *Ape*KI, the restriction enzyme *Mly*I was used (Hill et al., 2023). Sequencing was done on an Illumina HS4000 in pools of 96 at the UC Davis Genome Center (Davis, California).

### 2.5 SNP calling using BCF and VCFtools

Demultiplexed files were downloaded onto the NMSU computer Kraken (Penguin Relion XE1112 384 GiB). The reads were trimmed for quality, and adaptor sequences were removed using Cutadapt v4.1 (Martin, 2011). The trimmed reads were processed into a variant call format (VCF) file following the guidelines of the GB-eaSy pipeline (Wickland et al., 2017). The paired-end reads were aligned to the UCD10X genome (Hulse-Kemp et al., 2018) in parallel (Tange, 2018) using the Burrows Wheeler Aligner (BWA) (Li and Durbin, 2009). Packages BCFtools (Li, 2011) and VCFtools (Danecek et al., 2011) were used to call SNPs and filter for read depth, respectively. Insertions and deletions were ignored, and Single Nucleotide Polymorphism (SNPs) were called and then filtered to leave only SNPs with a minimum average read depth of 10.

### 2.6 Imputation and phasing using TASSEL

Each population was loaded into TASSEL (Bradbury et al., 2007), filtered by table sites with site min count = 80 (markers must have information for at least 80 individuals to be kept, 89.9%–97.5% of the complete populations), and then filtered by table taxa with min proportion of sites present = 0.3 (taxa must have 30% or more of marker information). After the filtering, remaining marker sites with missing data were imputed using the Linkage Disequilibrium k-Nearest Neighbors Imputation (LD KNNi) option with nearest neighbors = 5. The resulting genotype table was then transformed into ABH format using the ABH genotype option in TASSEL, which phases offspring genotypes given the genotypes of the parents.

### 2.7 QTL mapping with R/qtl

Genotype information from the TASSEL generated ABH files were read into R and combined with their matching phenotype information using the readCross function from R/qtl (v1.60; Broman et al., 2003). A series of filtering steps through R/qtl were also done to each pepper cross object. Duplicate markers were dropped, markers were filtered for segregation distortion, and duplicate F_2_ members and F_2_ members with unusually high numbers of crossover events were dropped. The genetic maps were also created using R/qtl, following the guidelines of Broman (2012) with an exception: instead of using functions formLinkageGroups and orderMarkers to put markers together on linkage groups and order them according to recombination fractions, markers were separated into linkage groups (chromosomes) and ordered along chromosomes according to the marker position on the physical map using the reference genome UCD v1.0 (Hulse-Kemp et al., 2018). This information was readily available because the markers were aligned to a reference genome. If a map showed a gap on a chromosome larger than 40 cM, then the markers were identified using iplotMap from the package qtlcharts (v0.16; Broman, 2015), and markers nearby those gaps were dropped. For each trait, a multiple-QTL model was found using stepwiseqtl, with the maximum number of QTLs set to the number of peaks seen in the summary plot of the logarithm of the odds (LOD) scores across the genome. This model was fit to the data using fitqtl, which reported LOD scores, p-value of chi-squared test, and percent variance explained, for both the model and individual QTL comprising the model. Additive and dominant effects were estimated using the effectscan function from R/qtl, and locations of QTLs were visualized using LinkageMapView (v2.1.2; Ouellette et al., 2018).

### 2.8 Statistical analysis

Correlations between phenotypes from all F_3_ families were found through the R package hmisc (v5.1-0; Harrell, 2023) and plotted with the R package corrplot (v0.92; Wei et al., 2021) in RStudio version 4.0.3 (R Core Team, 2020). Heritability estimates were done for the F_3_ families of each population using genotypes and phenotypes through the R package sommer (v3.4.2; Covarrubias-Pazaran, 2016) in RStudio. Statistics from detected QTLs were reported by the stepwiseqtl function in R/qtl (v1.60; Broman et al., 2003).

## 3 Results

Three F_2_ populations were created from one of three New Mexico pod–type parents, and a common other parent: a novel easy destemming parent derived from UCD-14, MUC14. For brevity in this section, they are referred to as Odyssey for the population from ‘NuMex Odyssey’ and MUC14, Iliad for the population from ‘NuMex Iliad’ and MUC14, and Joe E. Parker for the population from ‘NuMex Joe E. Parker’ and MUC14. Each population was created from reciprocal crosses between the parents. The F_2_ members of each population were grown in a greenhouse, their tissue was collected, DNA was extracted, and genotyping by sequencing (GBS) was carried out. The F_2_:F_3_ families of all the populations were grown out, and their fruit collected and measured. With phenotypic means of the F_3_ families representing the F_2_ parent genotype (**Table 1**), QTL analysis in R/qtl was done for the measurements destemming score, destemming force, fruit size index, fruit length, fruit width, and pericarp thickness. A handful of QTLs were found for each population, with a few QTLs having consensus between populations.

**Table 1:** Phenotypic means of parents and means and narrow-sense heritability estimates of F_3_ populations.

	Means of parents				Means of F_3_ families			Narrow-sense heritability of F_3_ families		
	MUC14	‘NuMex Odyssey’	‘NuMex Iliad’	‘NuMex Joe E. Parker’	‘NuMex Odyssey’ and MUC14	‘NuMex Iliad’ and MUC14	‘NuMex Joe E. Parker’ and MUC14	‘NuMex Odyssey’ and MUC14	‘NuMex Iliad’ and MUC14	‘NuMex Joe E. Parker’ and MUC14
Destemming force (N)	18.53 ± 3.60	47.91 ± 17.40	59.11 ± 13.94	33.9 ± 10.59	30.72 ± 8.78	30.00 ± 8.60	29.29 ± 8.91	0.77 ± 0.14	0.68 ± 0.14	0.78 ± 0.11
Destemming score	1.08 ± 0.50	2.43 ± 1.58	2.18 ± 0.51	1.87 ± 1.33	2.40 ± 0.76	1.98 ± 0.69	2.10 ± 0.66	0.48 ± 0.22	0.60 ± 0.17	0.56 ± 0.16
Pericarp thickness (mm)	1.81 ± 0.40	2.91 ± 0.72	3.37 ± 0.51	2.56 ± 0.51	2.60 ± 0.55	2.54 ± 0.48	2.49 ± 0.41	0.25 ± 0.17	0.13 ± 0.14	0.48 ± 0.16
Fruit length (cm)	6.02 ± 0.41	18.98 ± 2.17	18.86 ± 2.01	14.57 ± 1.48	9.26 ± 1.43	9.14 ± 1.29	8.96 ± 1.50	0.72 ± 0.15	0.63 ± 0.15	0.76 ± 0.12
Fruit width (cm)	2.35 ± 0.15	4.25 ± 0.47	4.50 ± 0.67	3.19 ± 0.38	2.74 ± 0.40	2.76 ± 0.40	2.82 ± 0.54	0.79 ± 0.13	0.67 ± 0.14	0.77 ± 0.15
Fruit size (index)	14.15 ± 1.62	81.02 ± 14.90	85.52 ± 18.27	43.02 ± 8.78	25.38 ± 5.31	25.24 ± 5.51	25.57 ± 7.69	0.72 ± 0.18	0.68 ± 0.14	0.82 ± 0.13

For the means of the parents and means of the F_3_ families, the standard deviation is shown. For heritability estimates, the standard error is shown.

### 3.1 GBS results

For each population, 107 DNA samples from the F_2_ generation were sent for GBS. The average value of Q30 for the sequences of the populations were 90.5, 91.0, and 91.6 for the Odyssey, Iliad, and Joe E. Parker populations, respectively, showing good sequence quality that was fairly consistent overall. After creating the variant calling files and filtering for depth, the Odyssey population had 3,384,423 markers, the Iliad population had 2,790,049 markers, and the Joe E. Parker population had 4,903,549 markers. After the filtering steps in TASSEL and in R/qtl described previously, the total number of markers decreased to 1,464, 2,613, and 8,254, respectively. The number of F_2_:F_3_ individuals in the phenotypic analysis was also reduced due to some plants not surviving to adulthood. There were 82 individuals in the Odyssey population, 84 in the Iliad population, and 89 individuals used in the Joe E. Parker population.

### 3.2 Genetic map construction and QTL analysis in R/qtl

For the Odyssey population, the map was constructed, and linkage analysis was done with 82 individuals and 1,464 markers (**Table 2**). The average chromosome length was 191.0 cM, with the largest being chromosome 1 with 287.1 cM and with the smallest being chromosome 10 with 93.1 cM. The map of the Iliad population was constructed with 93 individuals, and QTL linkage analysis was done with 84 individuals and 2,613 markers. The average chromosome length was 181.1 cM, with the largest being chromosome 6 with 292.9 cM and with the smallest being chromosome 5 with 126.6 cM. For the Joe E. Parker population, the map was built, and linkage analysis was done with 89 individuals and 8,254 markers. The average chromosome length was 238.4 cM, with the largest being chromosome 1 at 345.5 cM and with the smallest being chromosome 5 with 140.2 cM. All three populations had 12 linkage groups corresponding to the 12 chromosomes of *Capsicum annuum* (**Table 2**).

**Table 2:** Number of individuals and markers in each population used for QTL analyses and number of QTLs discovered.

Population	Number of individuals	Number of markers	QTLs detected	Total map length (cM)
‘NuMex Odyssey’ and MUC14	82	1,464	14	2,291.6
‘NuMex Iliad’ and MUC14	84	2,613	17	2,173.3
‘NuMex Joe E. Parker’ and MUC14	89	8,254	20	2,860.6

**Figure 1:**
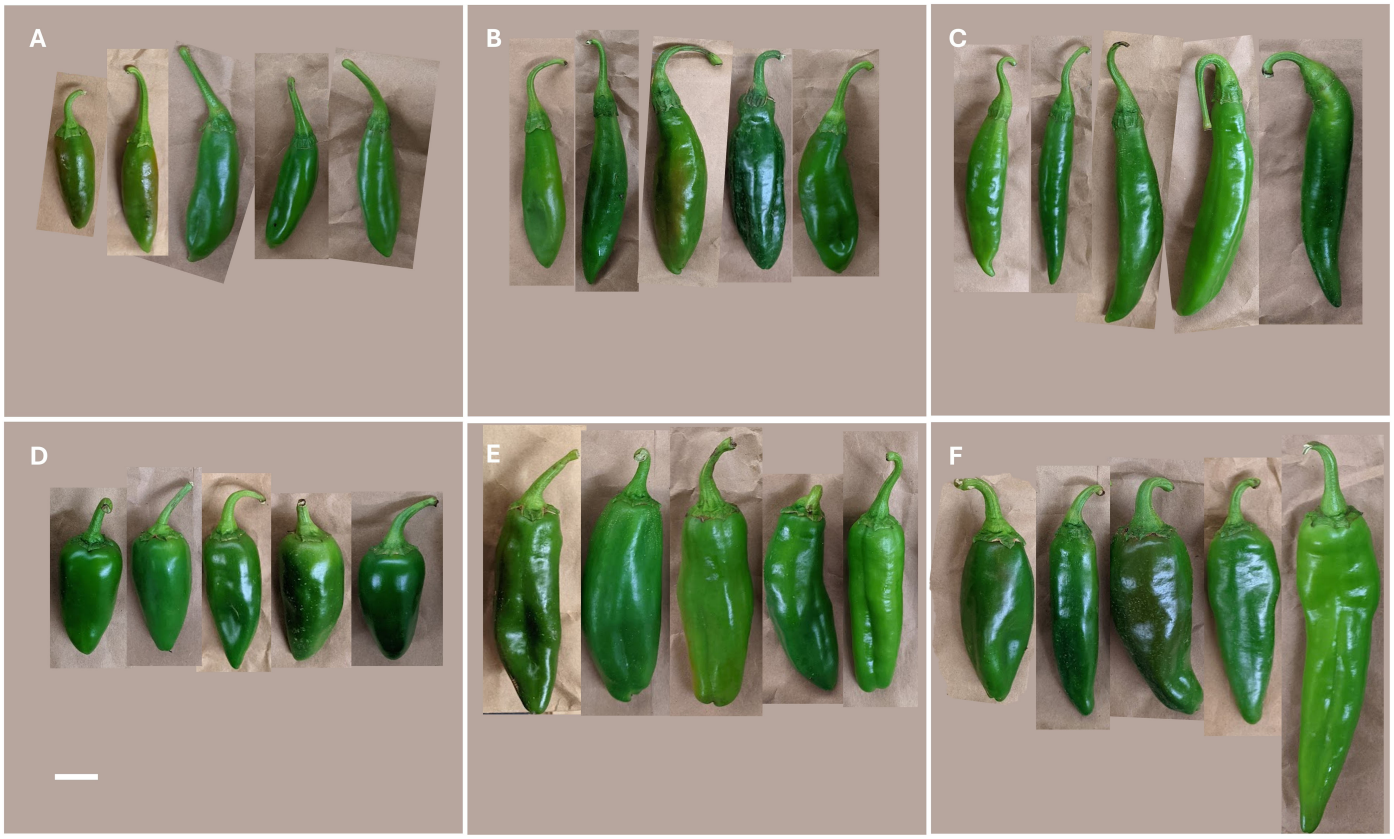
Fruit shape diversity seen in a selection of fruits from F_3_ families that came from all three populations created from MUC14 and a New Mexico pod–type parent. From these, some common fruit shapes observed were **(A)** oblong (most resembling MUC14), **(B)** oblong with narrow diameter at calyx attachment, **(C)** elongated, **(D)** conical, **(E)** rectangular, and **(F)** triangular (most resembling NM pod-type pepper) (USDA, 2015).

For all populations, QTL analysis was done on six traits. In total, there were five measurements collected, which all relate to traits that are important for breeding peppers compatible with mechanical harvesting. Two of these were measurements related specifically to the removal of the stem, and the other three were related to the size of the fruit. Another measurement, a fruit size index, was created by multiplying fruit width by fruit length. A variety of fruit sizes was seen among F_3_ families (**Figure 1**).

For destemming traits, QTLs were found. For destemming force, all three populations had major QTLs on chromosome 10 and two of the populations, Iliad and Joe E. Parker, identified additional major QTLs (PVE > 10%) (Collard et al., 2005) on chromosome 3 (**Table 3**). Similarly, for all three populations, QTLs on chromosome 10 were found for destemming score. In the Odyssey population, the marker at the peak of QTLs for destemming force and destemming score was the same. For the Odyssey and Iliad populations, QTLs on chromosome 5 were found for the destemming score phenotype. Other QTLs on chromosomes 1, 4, 6, 11, and 12 were identified for destemming score or force, but none were shared between populations except for the QTL on chromosome 6, which may be shared between the Iliad and Joe E. Parker populations (**Table 3**).

**Table 3:** Models for destemming QTLs discovered.

Model						QTL									
Population	Trait	Model	LOD	%Var	EST	Name	Chr	Map peak (cM)	Marker position (bp)	LOD	P-value (Chi^2^)	%Var	Additive effect	Dominant effect	1.5 LOD INT (cM)
‘NuMex Odyssey’ and MUC14	Destemming force	y ~ Q1 + Q2 + Q3 + Q1:Q2	23.3	73.0	31.5	odf1.1	1	12.1	11,303,171	1.5	3.30E−02	2.3	−1.47	−2.12	0–245.9
						odf5.1	5	15.5	11,878,217	5.4	0.00E+00	9.6	0.81	−3.58	8.3–21.7
						odf10.1	10	64.7	156,686,101	22.8	0.00E+00	69.9	−9.67	−0.66	59.6–66.2
										2.6	1.80E−02	4.2			
‘NuMex Iliad’ and MUC14	Destemming force	y ~ Q1 + Q2 + Q3 + Q1:Q2	24.4	73.8	29.9	idf3.1	3	223.4	252,158,660	9.9	0.00E+00	18.8	−4.18	2.76	217.6–235.5
						idf4.1	4	72.2	96,868,572	8.7	0.00E+00	16.1	−1.71	−2.09	0.0–153.1
						idf10.1	10	62.1	144,145,097	15.3	0.00E+00	34.5	−8.87	−0.59	59.7–63.2
										6.0	0.00E+00	10.3			
‘NuMex Joe E. Parker’ and MUC14	Destemming force	y ~ Q1 + Q2	21.2	66.5	29.2	jdf3.1	3	278.2	263,981,092	5.4	0.00E+00	10.7	−3.78	−1.61	272.0–282.1
						jdf10.1	10	66.6	144,655,980	19.3	0.00E+00	57.3	−9.09	−1.90	63.1–67.9
‘NuMex Odyssey’ and MUC14	Destemming score	y ~ Q1 + Q2 + Q3	12.0	49.1	2.5	ods1.1	1	256.0	242,243,485	3.7	0.00E+00	11.8	−0.30	−0.07	246.3–269.3
						ods5.1	5	39.6	19,317,986	5.4	0.00E+00	18.0	−0.24	0.46	36.7–53.8
						ods10.1	10	64.8	156,686,101	7.1	0.00E+00	25.0	−0.50	0.11	59.6–68.6
‘NuMex Iliad’ and MUC14	Destemming score	y ~ Q1 + Q2 + Q1:Q2	17.8	62.4	1.9	ids6.1	6	265.7	216,457,591	7.2	0.00E+00	18.3	0.25	−0.28	119.8–275.5
						ids10.1	10	59.7	138,159,084	15.4	0.00E+00	49.8	−0.61	−0.29	59.3–66.1
										4.4	0.00E+00	10.2			
‘NuMex Joe E. Parker’ and MUC14	Destemming score	y ~ Q1 + Q2 + Q3 + Q4	9.7	39.5	2.2	jds3.1	3	278.2	266,325,826	3.6	0.00E+00	5.0	0.13	−0.27	272.0–280.4
						jds6.1	6	29.4	7,685,400	3.4	0.00E+00	10.0	−0.21	0.29	23.8–219.9
						jds10.1	10	73.7	170,465,479	1.1	7.20E−02	4.8	−0.24	0.23	10.6–173.4
						jds11.1	11	31.3	15,460,412	1.9	1.40E−02	8.0	0.06	−0.42	7.7–271.7

Marker position is the physical position (bp) of the marker closest to the QTL peak. Percentage variance (%Var) is the percentage of phenotypic variance explained. For the estimated effect: positive values show that higher values are associated with MUC14, and negative values show that higher values are associated with the New Mexico pod–type parent (‘NuMex Odyssey,’ ‘NuMex Iliad,’ or ‘NuMex Joe E. Parker’).

For chile fruit traits, QTLs were found. For pericarp thickness, QTLs on chromosome 10 were found for all three populations nearby QTLs for destemming force and score. The Iliad and Joe E. Parker populations also shared major QTLs for pericarp thickness on chromosome 3. Three QTLs, two on chromosome 4 and one on chromosome 9 were found just in the Iliad population. For fruit size, QTLs were found in all populations on chromosomes 3 and Iliad and Joe E. Parker populations had QTLs on chromosomes 7, and 10. For fruit length, major QTLs on chromosomes 3 and 7 were found for all three populations. Fruit size and length QTLs were found on chromosome 7. These showed up at two positions: around 20 Mbp and 200 Mbp. The Odyssey population had a QTL around 20 Mbp for fruit length, whereas the population from Iliad had a QTL around 200 Mbp for fruit length and around 20 Mbp for fruit size. In the population from Joe E. Parker, a QTL at 20 Mbp was found for both fruit length and fruit size. An additional minor QTL on chromosome 9 was found only in the Joe E. Parker population. For fruit width, QTLs on chromosomes 10 and 3 were found for all populations with some population specific QTLs showing up on chromosomes 2, 4, and 9 (**Table 4**).

**Table 4:** Models for fruit size–related QTLs discovered.

Model						QTL									
Population	Trait	Model	LOD	%Var	EST	Name	Chr	Map peak (cM)	Marker position (bp)	LOD	P-value (Chi^2^)	%Var	Additive effect	Dominant effect	1.5 LOD INT (cM)
**‘NuMex Odyssey’ and MUC14**	Pericarp thickness	y ~ Q1	4.7	23.1	2.6	opt10.1	10	72.0	182,590,694	4.7	2.09E−05	23.1	−0.34	−0.19	71.3–77.1
**‘NuMex Iliad’ and MUC14**	Pericarp thickness	y ~ Q1 + Q2 + Q3 + Q4 + Q5 + Q1:Q2	21.1	68.5	2.5	ipt3.1	3	220.1	250,108,324	9.8	0.00E+00	22.4	−0.21	0.21	220.8–235.5
						ipt4.1	4	108.0	207,248,038	9.6	0.00E+00	21.7	0.00	−0.18	96.5–127.6
						ipt4.2	4	138.3	222,509,178	5.1	0.00E+00	10.3	−0.06	0.32	127.6–159.1
						ipt9.1	9	124.4	213,437,396	3.7	0.00E+00	7.1	−0.17	0.32	50.3–129.9
						ipt10.1	10	84.7	202,858,759	8.2	0.00E+00	17.9	−0.20	0.02	81.6–94.9
										5.4	0.00E+00	10.7			
**‘NuMex Joe E. Parker’ and MUC14**	Pericarp thickness	y ~ Q1 + Q2	9.6	39.1	2.5	jpt3.1	3	267.5	253,634,419	5.5	0.00E+00	19.9	−0.26	−0.10	261.6–274.4
						jpt10.1	10	68.5	156,686,101	4.8	0.00E+00	17.2	−0.24	−0.07	61.4–73.1
**‘NuMex Odyssey’ and MUC14**	Fruit length	y ~ Q1 + Q2	13.1	52.1	9.3	oflen3.1	3	166.7	256,169,734	9.1	0.00E+00	32.0	−0.94	0.13	166.7–175.3
						oflen7.1	7	109.0	22,578,707	8.6	0.00E+00	29.9	−0.89	−0.18	96.7–115.8
**‘NuMex Iliad’ and MUC14**	Fruit length	y ~ Q1 + Q2	8.9	38.7	9.3	iflen3.1	3	202.4	243,522,210	5.1	0.00E+00	19.6	−0.75	0.04	61.5–253.5
						iflen7.1	7	139.2	203,356,071	5.1	0.00E+00	19.8	−0.72	0.43	90.8–141.9
**‘NuMex Joe E. Parker’ and MUC14**	Fruit length	y ~ Q1 + Q2 + Q3 + Q4 + Q1:Q2	24.0	71.1	9.0	jflen3.1	3	89.2	37,884,203	4.8	1.00E−03	8.1	−0.29	−0.60	48.2–115.9
						jflen3.2	3	264.0	251,633,876	19.0	0.00E+00	48.3	−1.37	0.73	260.6–265.2
						jflen7.1	7	88.5	25,755,328	8.5	0.00E+00	15.8	−0.60	0.53	86.2–93.6
						jflen9.1	9	40.9	12,263,435	3.7	0.00E+00	6.0	0.11	1.05	35.7–41.9
										4.0	1.00E−03	6.7			
**‘NuMex Odyssey’ and MUC14**	Fruit width	y ~ Q1 + Q2 + Q3	19.3	66.1	2.8	ofwid3.1	3	58.7	45,036,332	6.7	0.00E+00	15.5	0.21	0.10	52.9–66.4
						ofwid3.2	3	156.7	247,806,932	6.8	0.00E+00	15.8	−0.12	0.21	149.5–159.6
						ofwid10.1	10	66.8	162,802,265	13.5	0.00E+00	38.6	−0.33	−0.16	59.6–70.7
**‘NuMex Iliad’ and MUC14**	Fruit width	y ~ Q1 + Q2	11.6	47.0	2.7	ifwid4.1	4	27.1	10,868,374	4.8	0.00E+00	15.7	−0.21	−0.18	13.3–41.9
						ifwid10.1	10	68.7	169,915,316	8.2	0.00E+00	29.9	−0.30	−0.13	63.2–70.9
**‘NuMex Joe E. Parker’ and MUC14**	Fruit width	y ~ Q1 + Q2 + Q3 + Q4 + Q3:Q4	27.2	75.6	2.8	jfwid2.1	2	43.4	76,586,271	5.5	0.00E+00	8.1	0.01	0.23	0.0–65.4
						jfwid3.1	3	267.5	253,634,419	10.3	0.00E+00	17.2	−0.31	0.01	263.5–274.4
						jfwid9.1	9	145.3	202,207,837	9.1	0.00E+00	14.7	−0.27	−0.10	97.4–165.1
						jfwid10.1	10	66.6	144,655,980	19.2	0.00E+00	41.6	−0.45	−0.23	66.2–67.2
										4.3	1.00E−03	6.2			
**‘NuMex Odyssey’ and MUC14**	Fruit size	y ~ Q1 + Q2	11.5	47.6	25.3	ofsiz3.1	3	149.5	247,171,453	8.5	0.00E+00	31.9	−3.99	2.56	148.9–156.7
						ofsiz11.1	11	73.9	38,130,299	3.9	0.00E+00	12.9	1.60	4.00	71.9–91.2
**‘NuMex Iliad’ and MUC14**	Fruit size	y ~ Q1 + Q2 + Q3 + Q1:Q3	14.0	53.5	26.0	ifsiz3.1	3	227.2	253,464,714	10.7	0.00E+00	37.0	−4.22	−0.73	223.4–235.5
						ifsiz7.1	7	56.0	20,881,290	2.8	2.00E−03	7.6	−1.31	0.96	0.0–181.4
						ifsiz10.1	10	94.9	207,525,158	7.0	0.00E+00	21.6	−2.65	−0.01	90.0–107.8
										1.5	1.33E−01	4.1			
**‘NuMex Joe E. Parker’ and MUC14**	Fruit size	y ~ Q1 + Q2 + Q3 + Q4 + Q2:Q3	27.6	76.0	26.6	jfsiz3.1	3	265.1	252,369,819	17.2	0.00E+00	34.3	−6.36	2.17	264.0–269.8
						jfsiz7.1	7	87.8	22,578,707	10.1	0.00E+00	16.5	−1.57	2.19	76.7–93.6
						jfsiz9.1	9	159.7	212,178,108	7.0	0.00E+00	10.5	−4.01	−0.07	40.4–166.7
						jfsiz10.1	10	66.6	144,655,980	8.8	0.00E+00	13.8	−3.70	−2.84	63.1–73.1
										4.1	1.00E−03	5.6			

Marker position is the physical position (bp) of the marker closest to the QTL peak. Percentage variance (%Var) is the percentage of phenotypic variance explained. For the estimated effect: positive values show that higher values are associated with MUC14, and negative values show that higher values are associated with the New Mexico pod–type parent (‘NuMex Odyssey,’ ‘NuMex Iliad,’ or ‘NuMex Joe E. Parker’).

**Figure 2:**
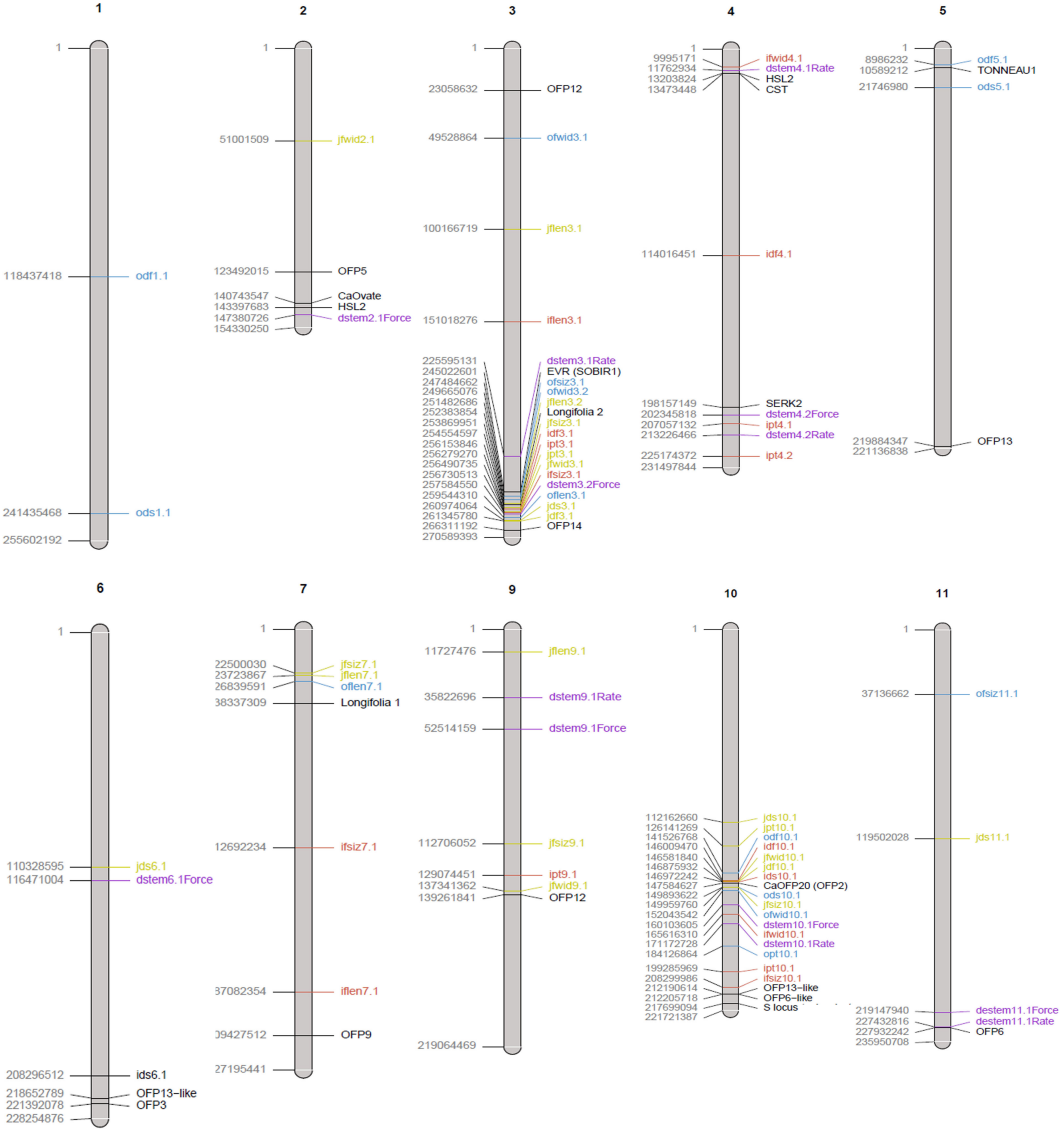
Physical map of QTLs reported in this study from the three biparental populations ‘NuMex Iliad’ and MUC14 (red), ‘NuMex Odyssey’ and MUC14 (blue), and ‘NuMex Joe E. Parker’ and MUC14 (yellow). QTLs previously reported from Hill et al. (2023) (purple), and genes of interest related to fruit size or abscission (black) are also shown. The location of the QTL is plotted approximately in the center of its confidence interval. Genes are plotted at their starting location. Chromosome length is according to the UCD v1.0 genome. Chromosomes on which no QTLs were found in this study were excluded (chromosomes 8 and 12).

**Figure 3:**
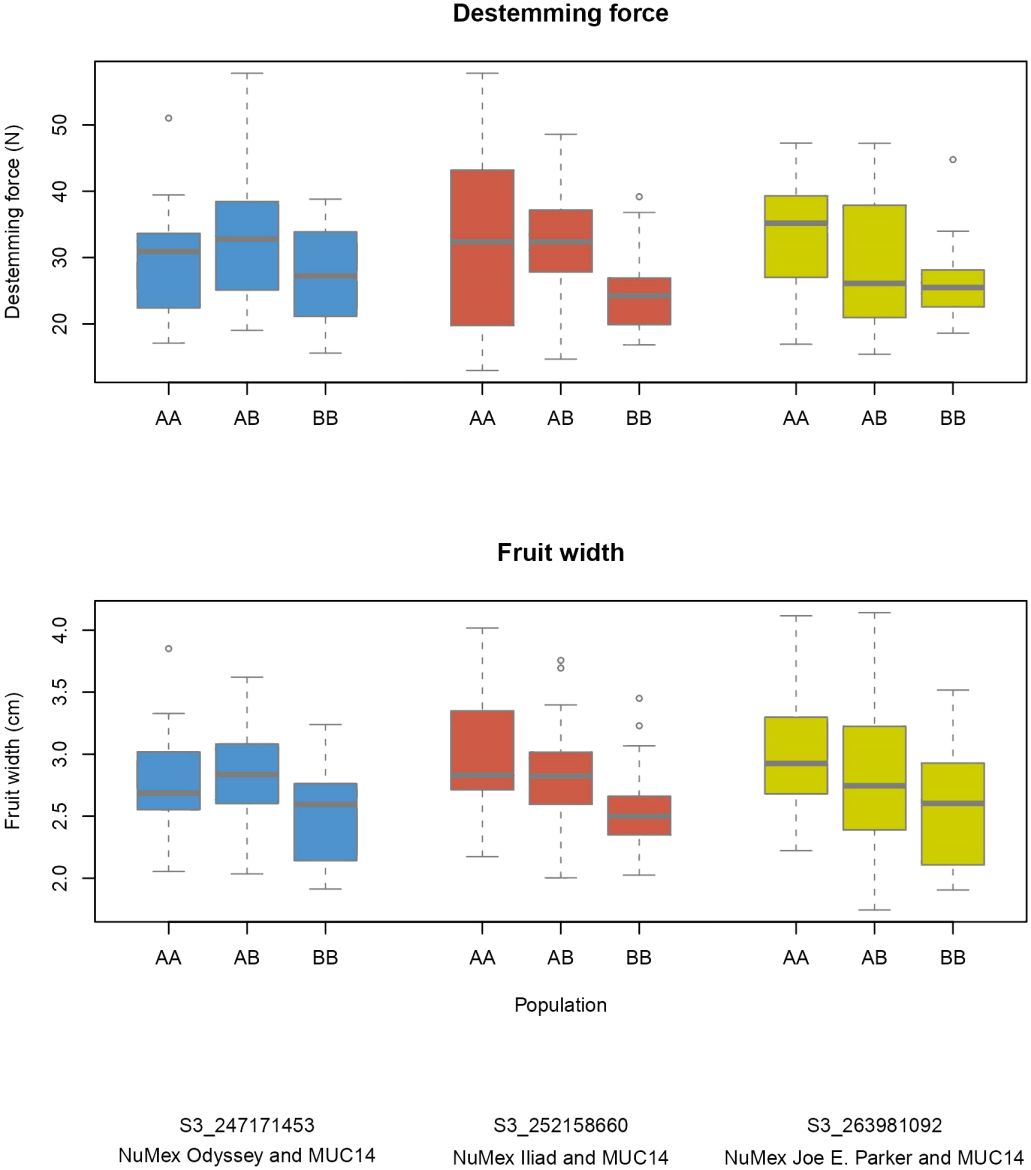
Phenotype by genotype boxplots for QTLs of destemming force and fruit width on chromosome 3. Labeled as AA is the genotype of the New Mexico pod–type parent (NuMex Odyssey, NuMex Iliad, and NuMex Joe E. Parker), and BB is the genotype of MUC14.

**Figure 4:**
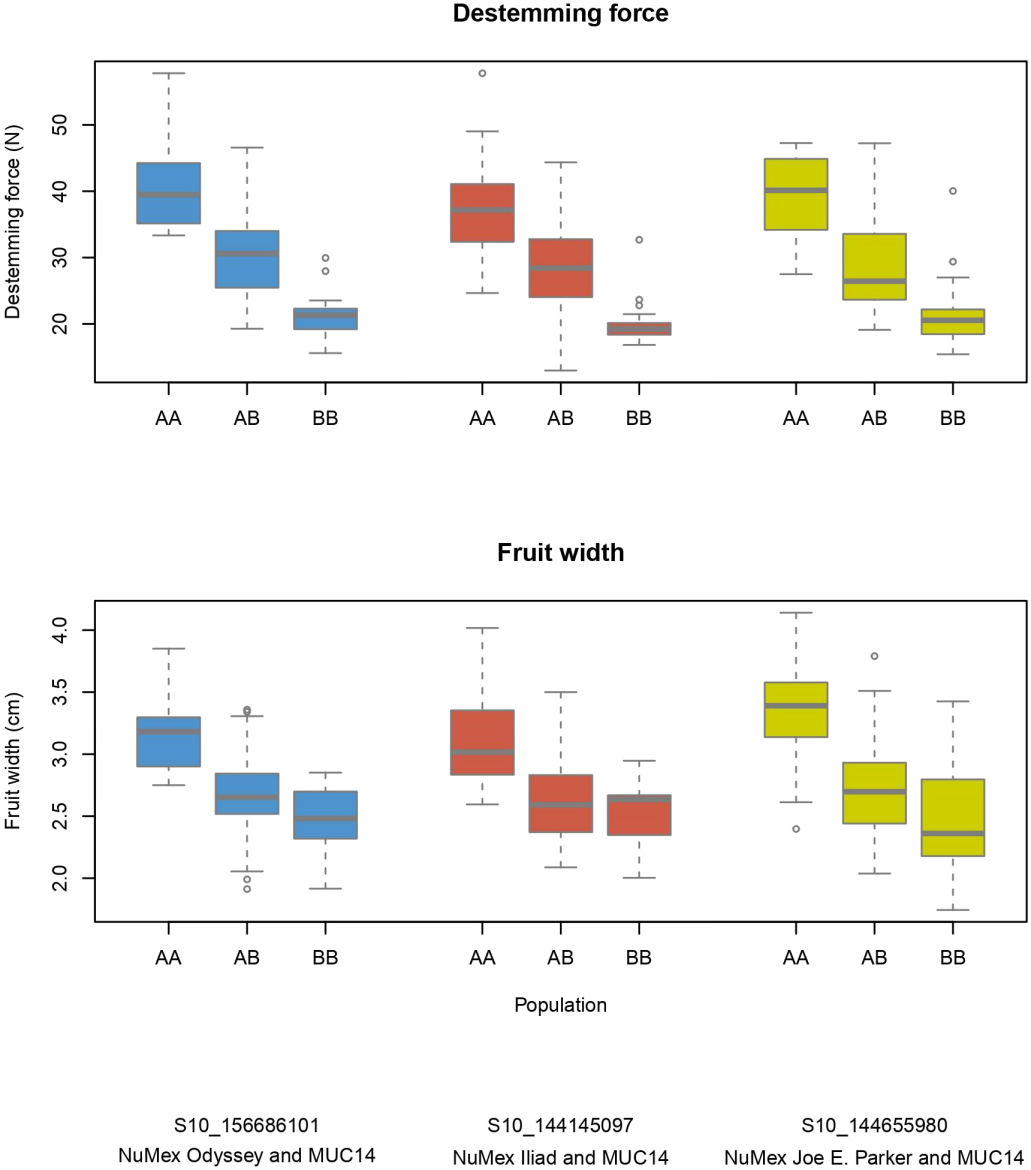
Phenotype by genotype boxplots for QTLs of destemming force and fruit width on chromosome 10. Labeled as AA is the genotype of the New Mexico pod–type parent (NuMex Odyssey, NuMex Iliad, and NuMex Joe E. Parker), and BB is the genotype of MUC14.

Several QTLs for different traits were found nearby one another on the chromosomes. Fruit length, width, size, pericarp thickness, destemming score, and destemming force QTL peaks were seen around the same area on chromosome 3 (**Figure 2**). Markers closest to QTL peaks around this area on chromosome 3 were clustered around 243–263 Mbp. For the Iliad and Joe E. Parker populations, size QTL peaks were around 20 Mbp on chromosome 7. Similarly, length QTL peaks for the Odyssey and Joe E. Parker populations are seen close together around 20 Mbp on chromosome 7. Width and size QTL peaks from the Joe E. Parker population and pericarp thickness from the Iliad population were clustered around 213 Mbp on chromosome 9. Strikingly, QTLs for destemming force, destemming score, fruit width, fruit size, and pericarp thickness were scattered along the lower half of chromosome 10 (**Figure 2**). When the phenotype was plotted by genotype, the general pattern seen was that the more alleles at QTLs on chromosomes 3 and 10 an F_2_ individual had from MUC14, the lesser destemming force was and the less wide the fruit was in the F_3_ family (**Figure 3** and **Figure 4**).

### 3.3 Fruit trait correlations using hmisc

**Figure 5:**
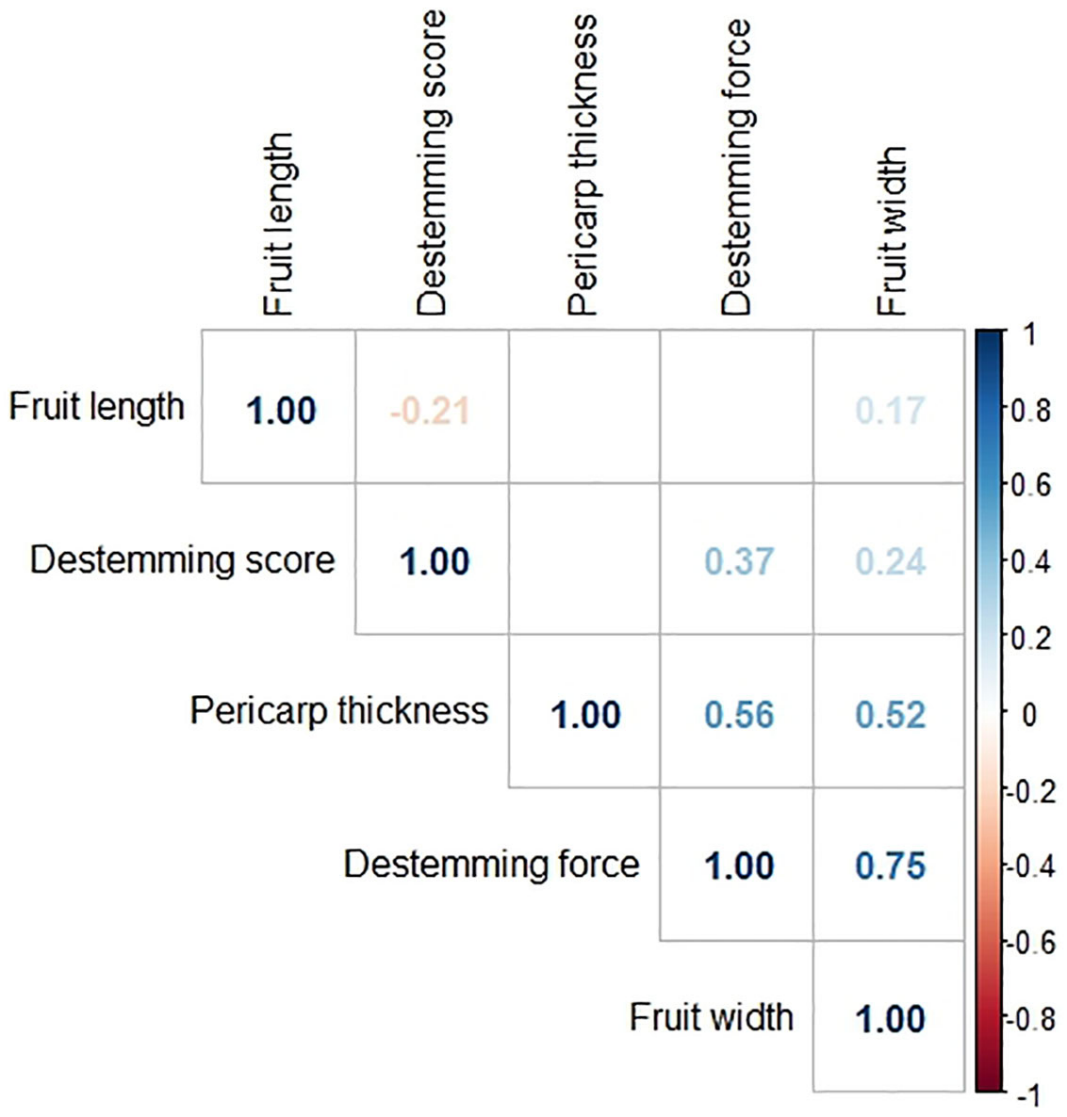
Correlation matrix among phenotypes of the F_3_ family means for all populations. Pearson’s correlation coefficients are shown for trait pairs with significant correlations (p-value threshold=0.05).

Pearson’s correlation coefficients and their p-values were found between each pair of phenotypes. The phenotype pair with the strongest relationship, which was also significant was between destemming force and fruit width (*r* = 0.75, *p* = 0.00e+00), followed by pericarp thickness and destemming force (*r* = 0.56, *p* = 0.00e+00) and pericarp thickness and fruit width (*r* = 0.52, *p* = 0.00e+00). Pairs with a moderately positive relationship were between destemming score and destemming force (*r* = 0.37, *p* = 2.09e−10), destemming score and fruit width (*r* = 0.24, *p* = 4.69e−05), and fruit width and fruit length (*r* = 0.17, *p* = 3.39e−03). One pair, fruit length and destemming score, had a negative relationship (*r* = −0.21, *p* = 3.04e−04). The pairs fruit length and destemming force, fruit length and pericarp thickness, and destemming score and pericarp thickness did not have significant correlation coefficients (*p*-values are 5.24e−01, 2.59e−01, and 3.81e−01, respectively) (**Figure 5**).

## 4 Discussion

Implementation of mechanical harvesting for green chile is a goal of the industry in the United States. Crucial for this is the development of varieties with traits that facilitate the process of mechanical harvesting. One such important trait is easy destemming: because the green chile pepper’s woody pedicel is inedible, it is usually removed by hand in the field. For mechanical harvesting, varieties in which pedicels can be removed completely with little force applied would be ideal. This study sought to trace the genetic source of a novel easy destemming trait in crosses between a semi-domesticated UCD-14 derived pepper, MUC14, and several New Mexico pod–type peppers. All three biparental F_2_ populations were genotyped using GBS, F_3_ families were grown, and destemming and fruit measurements were taken from harvested F_3_ fruit. For the three populations studied, MUC14 was crossed to ‘NuMex Odyssey,’ ‘NuMex Iliad,’ and ‘NuMex Joe E. Parker,’ and QTL analyses were performed, discovering 14, 17, and 20 QTLs, respectively, for six traits. For several of these QTLs, consensus between populations, as well as overlap between QTLs for different traits, was observed.

One other study has searched for destemming force QTLs in chile pepper. Studying populations that came from the easy destemming Mexican landrace UCD-14, Hill et al. (2023) reported destemming force QTLs on chromosomes 2, 3, 4, 9, 10, and 11. In the present study, QTLs were found close to theirs on chromosomes 3, 4, and 10. In this study on chromosome 3, fruit length, fruit width, fruit size, pericarp thickness, destemming force, and destemming score QTLs were detected nearby where they found pull force and force QTLs (**Figure 2**). In this region, they point out a candidate gene, Mitogen-activated protein kinase kinase 5 (*MKK5*), that is involved in the abscission signal transduction pathway in *Arabidopsis thaliana* (Cho et al., 2008). This is notable as they suggest easy destemming of UCD-14 could come from the activation of an abscission zone at the pedicel/fruit boundary. On one end of chromosome 4, a pericarp thickness QTL in this study and destemming force and rate QTLs in theirs were found nearby one another. On the other end of chromosome 4, a fruit width QTL in this study and a destemming rate QTL in theirs were nearby one another. These are close to the genes *CST* and *SERK2*, which are also related to abscission (Burr et al., 2011; Meng et al., 2016) (**Figure 2**). Finally, on chromosome 10, we found destemming force, destemming score, fruit width, fruit size, and pericarp thickness QTLs nearby where they found destemming force, rate, and pull rate QTLs (**Figure 2**).

In addition to destemming traits, this study measured fruit size. In pepper, there are many reports of QTLs for fruit size traits being found clustered together on chromosome 3 (Hill et al., 2017; Lozada et al., 2021). In an early QTL study that covered fruit traits in pepper, fruit diameter, fruit length, fruit shape, and pericarp thickness were all found to have QTLs in an overlapping region on chromosome 3 (Chaim et al., 2001). For an interspecific cross between *C. annuum* and *C. frutescens*, QTLs for the fruit traits maximum width, maximum height, width mid area, and fruit area, were found together in a region on their map between 54.8 cM and 100 cM on chromosome 3 (Yarnes et al., 2013). A double haploid *C. annuum* study found QTLs for fruit length, fruit diameter, and fruit shape at the same marker on chromosome 3 (Mimura et al., 2012). For a *C. annuum/C. annuum* cross, QTLs for fruit traits—fruit length, fruit diameter, and fruit shape—were found concentrated on chromosome 3, mostly in the range between 107 Mbp and 252 Mbp (Han et al., 2016). Similarly, fruit longitude and fruit diameter in a population derived from the blocky sweet pepper Maor and pungent disease resistant CM334 (both *C. annuum*) found QTLs for these traits within a region on chromosome 3 between 227 Mbp and 247 Mbp in the CM334 genome (Chunthawodtiporn et al., 2018). These two latter studies both have a physical range on the genome where regions of QTLs on chromosome 3 from this study overlap (**Supplementary Table S1**).

A couple of genes have been proposed to underlie the previously discovered fruit size QTLs on pepper chromosome 3. A SNP marker at 183,386,147 bp in the CM334 genome was found within a gene, *Longifolia 1-like*, that was homologous to *lng1* (LONGIFOLIA1) and *lng2* (LONGIFOLIA2), genes that encode TONNEAU1-recruiting motif (TRM) proteins, which are known to promote cell elongation in the model organism *Arabidopsis thaliana* (Zhang et al., 2020). In pepper, this location was related to fruit shape, determining whether the fruit is more circular or oval shaped (Colonna et al., 2019). Although our chromosome 3 QTLs do not overlap with this region, the predicted gene *Longifolia 2* (LOC107862335) sitting at 252,383,854–252,389,858 bp in the UCD v1.0 genome, is nearby our QTLs. Another gene proposed to regulate cell division is *CaARF2-i2*, which is on chromosome 3 at 244,772,375–250,606,405 bp in the CM334 genome (Hill et al., 2017).

There is also precedence for fruit size QTLs in pepper on chromosome 10. In one study, fruit shape, fruit diameter, and pericarp thickness QTLs were all reported around a QTL called *fs10.1* on chromosome 10 responsible for fruit shape (Chaim et al., 2001, Chaim et al., 2003). Recently, a multi-environment GWAS done for a *C. annuum* collection found *FShI-P10.1*, a fruit shape index QTL, and *FLe-P10.1*, a fruit length QTL, on chromosome 10 in all locations that they studied (McLeod et al., 2023). For fruit size traits measured through tomato analyzer—transverse perimeter and transverse area—QTLs were found on chromosome 10 for RILs from a cross between two *C. annuum* parents (Chunthawodtiporn, et al., 2018). Fruit width and diameter QTLs on chromosome 10 were also reported by Eggink et al. (2013) and Mimura et al. (2012). All of these, except for Chaim et al. (2003), reported their QTL for this area as accounting for about 10% of variance for their respective trait. In this study, when fruit size is broken down into its components, fruit length and width, QTLs on chromosome 10 are only found for fruit width in all three populations, accounting for a much greater percent variance explained (29%–47%). This is more similar to results from Chaim et al. (2003) who reported percent variance explained for fruit shape of this QTL to be 30%–44% (**Supplementary Table S2**).

An early mutagenesis study hinted that the gene underlying *fs10.1* was likely creating variation in overall fruit size through a change in cell shape (Borovsky and Paran, 2011). Later, this research group identified through bulked segregant analysis using RNA sequencing of F_2_ ovary tissue that the gene underlying *fs10.1* is an ortholog of the tomato suppressor of ovate family proteins (OFPs), *SlOFP20*, in pepper named *CaOFP20* (Borovsky et al., 2022). When *CaOFP20* was silenced, knockdown fruits appeared elongated compared to control fruits, suggesting that *CaOFP20* acts to repress fruit elongation. OFPs have been implicated in fruit shape in land plants like *Arabidopsis* (Wang et al., 2011) and tomato (Liu et al., 2002) and have been identified in shaping pepper fruit size/weight before (Tsaballa et al., 2011). OFPs are thought to confer this effect in part though interactions with TRM proteins, which impacts the formation of the pre-prophase band during cell division (Snouffer et al., 2020). *CaOFP20* (LOC107845902) is found on the UCD v1.0 genome on chromosome 10 at 147,584,627–147,586,245 bp. This is nearby major fruit width, destemming force, and destemming score related QTLs found close together on chromosome 10 found for all three populations in this study. Moreover, in a genome-wide analysis, Luo et al. (2022) searched for OFPs throughout the *Capsicum annuum* genomes of Zunla-1, CM334, and Chiltepin, finding 8, 5, and 7 OFP genes (respectively) residing on a distal end of chromosome 10. The OFPs “CaOFP15-21” in their study were found in tandem repeats on chromosome 10 and showed similar expression in different tissues. The redundancy of OFPs on chromosome 10 in pepper may explain the span of positions for fruit size–related QTLs in this region.

Breeding for easy destemming may be complicated by the connection between destemming force and fruit size. Fruit size and shape are a fundamental part of the appeal of pepper varieties, so selecting for traits that are closely linked to genetic sources of fruit variability can be a challenge (Naegele et al., 2014). In this study, significant positive correlations were seen between destemming force and the fruit traits pericarp thickness and fruit width (**Figure 5**). Additionally, the largest QTLs for destemming force were found at the two high-density QTL regions for fruit traits on chromosomes 3 and 10 (**Figure 2**). Plotting destemming force and fruit width measurements alongside one another at the markers closest to QTL peaks on chromosomes 3 and 10 displays this relationship, as similar patterns of changing phenotype according to genotype can be seen for both traits (**Figure 3** and **Figure 4**). This could be the result of pleiotropy or linkage and may complicate breeding for easy destemming while maintaining a New Mexico pod–type.

## 5 Conclusion

Easy destemming in chile pepper appears to be intertwined with fruit size. In particular, the region on chromosome 10 between 138 Mbp and 211 Mbp holds QTLs, which account for a large percentage variance for both fruit width and destemming force. This same region is occupied by OFPs, a gene family that is known to mediate fruit shape in several species, including tomato, and the gene *CaOFP20* in pepper is documented as having a dramatic effect on pepper fruit elongation when knocked down. It is thought that OFPs modify fruit shape through interactions with TRMs. Two out of three populations had destemming force QTLs, and all three populations had fruit size QTLs, on chromosome 3 around 247–264 Mbp. In this region, there is a TRM called *Longifolia 2*, a homolog of a gene in *Arabidopsis* that mediates cell elongation. These areas on chromosomes 3 and 10 have been implicated in a separate destemming force study in California.

It is possible that genes influencing fruit shape are nearby other genes that are contributing to the easy destemming trait. For example, in the region of interest on chromosome 3, there is a homolog of a gene (*EVR*), which can contribute to floral organ abscission in *Arabidopsis* (Leslie et al., 2010). At this point, it is not clear whether the fruit shape genes implicated in this study are solely responsible for easy destemming or if it is connected to contributing genes through linkage. Either way, future breeding efforts toward easy destemming in pepper will require consideration of fruit size and shape.

## Data availability statement

The datasets presented in this study can be found in online repositories. The names of the repository/repositories and accession number(s) can be found below: https://www.ebi.ac.uk/eva/, PRJEB70773.

## Author contributions

FO: Data curation, Formal analysis, Investigation, Visualization, Writing – original draft, Writing – review & editing. TH: Conceptualization, Funding acquisition, Writing – review & editing. AD: Conceptualization, Funding acquisition, Writing – review & editing. AG-L: Investigation, Writing – review & editing. SW: Funding acquisition, Resources, Supervision, Writing – review & editing.

## Supplementary material

Supplementary Table 1 Previously reported chile pepper fruit size QTLs on chromosome 3.

Supplementary Table 2 Previously reported chile pepper fruit size QTLs on chromosome 10.
